# Characterization of the emergent properties of a synthetic quasi-cellular system

**DOI:** 10.1186/1471-2105-13-S4-S9

**Published:** 2012-03-28

**Authors:** Lorenzo Lazzerini-Ospri, Pasquale Stano, PierLuigi Luisi, Roberto Marangoni

**Affiliations:** 1 Dipartimento di Informatica, Università di Pisa, L.go B. Pontecorvo 3, 56127 Pisa, Italy; 2 Dipartimento di Biologia, Università di Roma III, Via G. Marconi 446, 00146 Roma, Italy; 3 Istituto di Biofisica del CNR, Via G. Moruzzi 1, 56124 Pisa, Italy

## Abstract

**Background:**

The process of solutes entrapment during liposomes formation is interesting for the investigation of the relationship between the formation of compartments and the distribution of molecules inside them; a relevant issue in the studies of the origin of life. Theoretically, when no interactions are supposed among the chemical species to be entrapped, the entrapment is described by a standard Poisson process. But very recent experimental findings show that, for small liposomes (100 nm diameter), the distribution of entrapped molecules is best described by a power-law function. This is of a great importance, as the two random processes give rise to two completely different scenarios. Here we present an *in silico *stochastic simulation of the encapsulation of a cell-free molecular translation system (the PURE system), obtained following two different entrapment models: a pure Poisson process, and a power-law. The protein synthesis inside the liposomes has been studied in both cases, with the aim to highlight experimental observables that could be measured to assess which model gives a better representation of the real process.

**Results:**

Firstly, a minimal model for in vitro protein synthesis, based on the PURE system molecular composition, has been formalized. Then, we have designed a reliable experimental simulation where stochastic factors affect the reaction course inside the compartment. To this end, 24 solutes, which represent the PURE system components, have been stochastically distributed among vesicles by following either a Poisson or a power-law distribution. The course of the protein synthesis within each vesicle has been consequently calculated, as a function of vesicle size. Our study can predict translation yield in a population of small liposomes down to the attoliter (10^-18 ^L) range. Our results show that the efficiency of protein synthesis peaks at approximately 3·10^-16 ^L (840 nm diam.) with a Poisson distribution of solutes, while a relative optimum is found at around 10^-17 ^L (275 nm diam.) for the power-law statistics.

**Conclusions:**

Our simulation clearly shows that the wet-lab measurement of an effective protein synthesis at smaller volumes than 10^-17 ^L would rule out, according to our models, a Poisson distribution of solutes.

## Background

The origin of life is a scientific problem not outside the experimental realm.

The scientific consensus holds that life on this planet emerged through a process of gradual, incremental, chemical evolution from non-living matter, a process known as abiogenesis [1,2].

In the last decades, a great effort has been done to reconstruct, at least partially, the physical-chemical steps leading to the origin of life on our planet. A definitive proof of concept would be provided by the *ex novo *assembly of a living system - that is, a membrane-bound structure characterized by self-replication and self-maintenance, capable of Darwinian evolution [3,4].

The eventual synthetic pathway would also help answer questions concerning the relative importance of chance and necessity in the historical origin of life on earth [5,6].

Ideally, an experimental approach should start from the simplest allegedly prebiotic chemical precursors. Recently, however, an alternative approach, called "semi-synthetic", has been proposed. This seems not only well focused to provide the proof of principle that living forms might indeed emerge from the self-organization of their molecular components, but also appears experimentally feasible [4,7,8].

Liposomes, as closed spherical bilayers, are the most obvious candidates as cell compartments. Their key properties, subject to ongoing research, range from autopoiesis, or self-assembly from amphiphilic precursors in water [9-11], to their ability to incorporate various molecules, to their growth and division at this expenses of free precursors [12-17].

In order to build liposome-based minimal cells, it is necessary to encapsulate the minimal number of molecules such as proteins and nucleic acids in order to accomplish the essential process typical of cellular life. Current research focuses on the synthesis of functional proteins inside liposomes. The translation "module", in fact, is one of the most complex and important for cellular metabolism. Therefore, its implementation in liposome-based systems not only allows the production of proteins (which, in turn, act as catalysts or structural components of the synthetic cell, extending the repertoire of the available functions), but also well models the complexity of living cells.

Several protein expression systems have been successfully encapsulated inside liposomes [18-22] (for a review, see [8]). So far, these systems have been used to express the Green Fluorescent Protein (GFP), an easily detectable protein, and few other enzymes. The final goal of this approach is the intraliposomal expression of enzymes that ultimately catalyze the regeneration of all internalized and membrane-forming components, so that a coordinated "core-and-shell" self-reproduction can be achieved [4,23].

**Figure 1:**
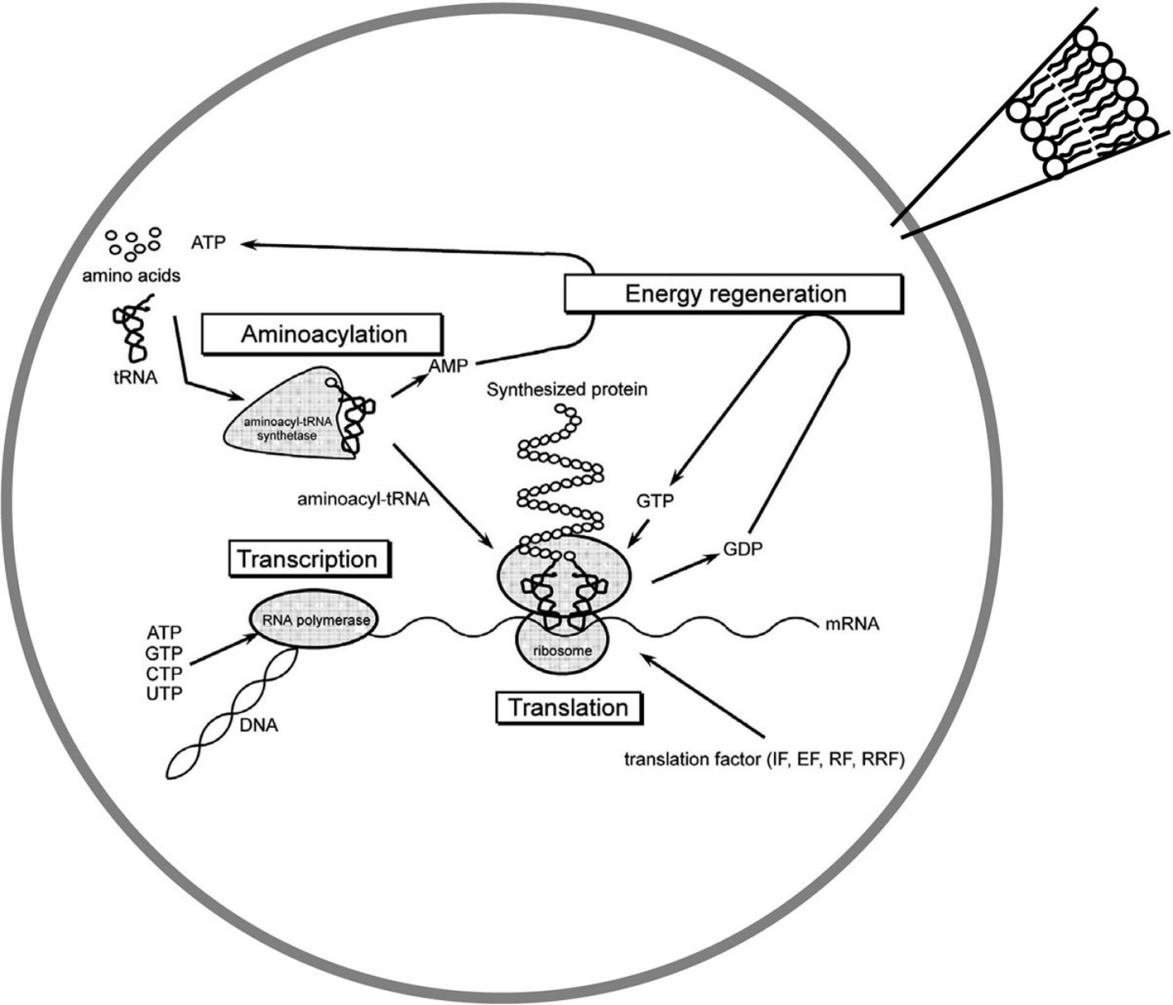
**The PURE system: main metabolic blocks**. A schematic drawing of the metabolic pathways included in the PURE system and hosted in a functionalized liposome (reproduced from [42] with permission from Springer, and from [35] with permission from Elsevier).

Our focus was on those approaches where a particular translation apparatus called the PURE system is encapsulated inside lipid vesicles (see Figure 1).

The PURE system (Protein synthesis Using Recombinant Elements) was developed by Ueda and colleagues [24] and is now commercially available. It comprises about 80 different macromolecular species (plus amino acids, nucleotides, etc.), representing the minimal set of the *E. coli *translational machinery (see Table 1). Thirty-six of these macromolecular species have been purified individually by Ni/His6 affinity chromatography after overexpression in *E. coli *cells. Ribosomes are specifically isolated by sucrose-density-gradient centrifugation. By mixing in proper ratios the isolated compounds, the *E. coli *translation activity is reconstituted effectively [24] and can be used to synthesize a protein starting from an encoding DNA (or RNA) sequence. As evident in Figure 1, the PURE system is indeed a modular tool that recapitulates in vitro the following metabolic processes: (i) aminoacylation of tRNAs; (ii) translation (initiation, elongation, termination); (iii) regeneration of the energetic species (ATP, GTP); and, optionally, (iv) transcription.

**Table 1:** PURE system components*

Component	**Conc**.	Component	**Conc**.
Translation factors		ProRS	1,300 U/mL
IF1	2.7 μM	SerRS	1,900 U/mL
IF2	0.4 μM	ThrRS	1,300 U/mL
IF3	1.5 μM	TrpRS	630 U/mL
EF-G	0.26 μM	TyrRS	630 U/mL
EF-Tu	0.92 μM	ValRS	3,100 U/mL
EF-Ts	0.66 μM		
RF1	0.25 μM	Other enzymes	
RF2	0.24 μM	MTF	4,500 U/mL
RF3	0.17 μM	Ribosomes	1.2 μM
RRF	0.5 μM	Creatine kinase	4 μg/mL
		Myokinase	3 μg/mL
Amino acyl-tRNA synthetases (RSs)		Nucleoside diphosphate kinase	1.1 μg/mL
AlaRS	1,900 U/mL	Pyrophosphatase	2 U/mL
ArgRS	2,500 U/mL	T7 RNA polymerase	10 μg/mL
AsnRS	20 mg/mL		
AspRS	2,500 U/mL	Energy sources	
CysRS	630 U/mL	ATP	2 mM
GlnRS	1,300 U/mL	GTP	2 mM
GluRS	1,900 U/mL	CTP	1 mM
GlyRS	5,000 U/mL	UTP	1 mM
HisRS	630 U/mL	Creatine phosphate	20 mM
IleRS	2,500 U/mL		
LeuRS	3,800 U/mL	Other components	
LysRS	3,800 U/mL	20 amino acids	0.3 mM
MetRS	6,300 U/mL	10-formyl-5,6,7,8-tetrahydrofolic acid	10 mg/mL
PheRS	1,300 U/mL	tRNAmix (Roche)	56 Abs260

*The components are solubilized in 50 mM HEPES-KOH pH 7.6; 100 mM potassium glutamate, 13 mM magnesium acetate, 2 mM spermidine, 1 mM DTT. One unit of activity was defined as the amount of enzyme that catalyzes the formation of 1 pmol of amino acyl-tRNA in 1 min (reproduced from [35] with permission from Elsevier). The conversion from U/mL to μM has been taken from [27].

Protein-expressing vesicles have been created by encapsulating the PURE system components inside liposomes [25-30]. Water-soluble proteins as GFP, Qβ-replicase, β-galactosidase, have been effectively expressed inside lipid vesicles of diverse lipid compositions, sizes and morphologies prepared by a variety of methods. Membrane proteins have also been expressed in specially designed liposomes [28].

Experiments are usually done by preparing liposomes in an aqueous solution containing the PURE system. The dispersion of lipids in water can be carried out by different methods, for example by swelling preformed thin lipid films, or by rehydrating freeze-dried liposome membranes, or also by injecting a small aliquot of a lipid-containing alcoholic solution. Consequent to lipid dispersion in the aqueous phase, liposomes form spontaneously. It is during this self-organization step that the PURE system components, floating in solution, are entrapped in the liposome's inner space. Non-entrapped PURE system components are generally inhibited from reacting by the addition of EDTA, RNAses, or proteases, so that protein synthesis occurs only inside liposomes.

In order to produce a realistic *in silico *model of intraliposomal translation, both solute entrapment and protein synthesis must be simulated stochastically. In fact, it is evident that stochastic effects will affect the solute encapsulation efficiency especially in the case of small liposomes and low solute concentrations. It is generally accepted that the physics of water-soluble solute encapsulation follows a Poisson statistics, where the mean number μ_i _of entrapped i-th solute (initially present in solution with a concentration C_i_) in a vesicle of volume V is given by μ_i _= N_A _C_i _V (N_A _being the Avogadro number). Fluctuations around this mean value can be described by the Poisson distribution, and are responsible for the fact that the true outcome of the encapsulation step is actually a population of vesicles of different solute content (in terms of number of chemical species and their amount). Furthermore, even independently of this effect, protein synthesis can display a stochastic behaviour when occurring in small volumes with a low number of solutes, as is the case in attoliter (10^-18 ^L) containers. This behaviour is dependent on compartment size, because translation consists of higher-than-first order reactions whose likelihood depends, *inter alia*, on reaction volume.

**Figure 2:**
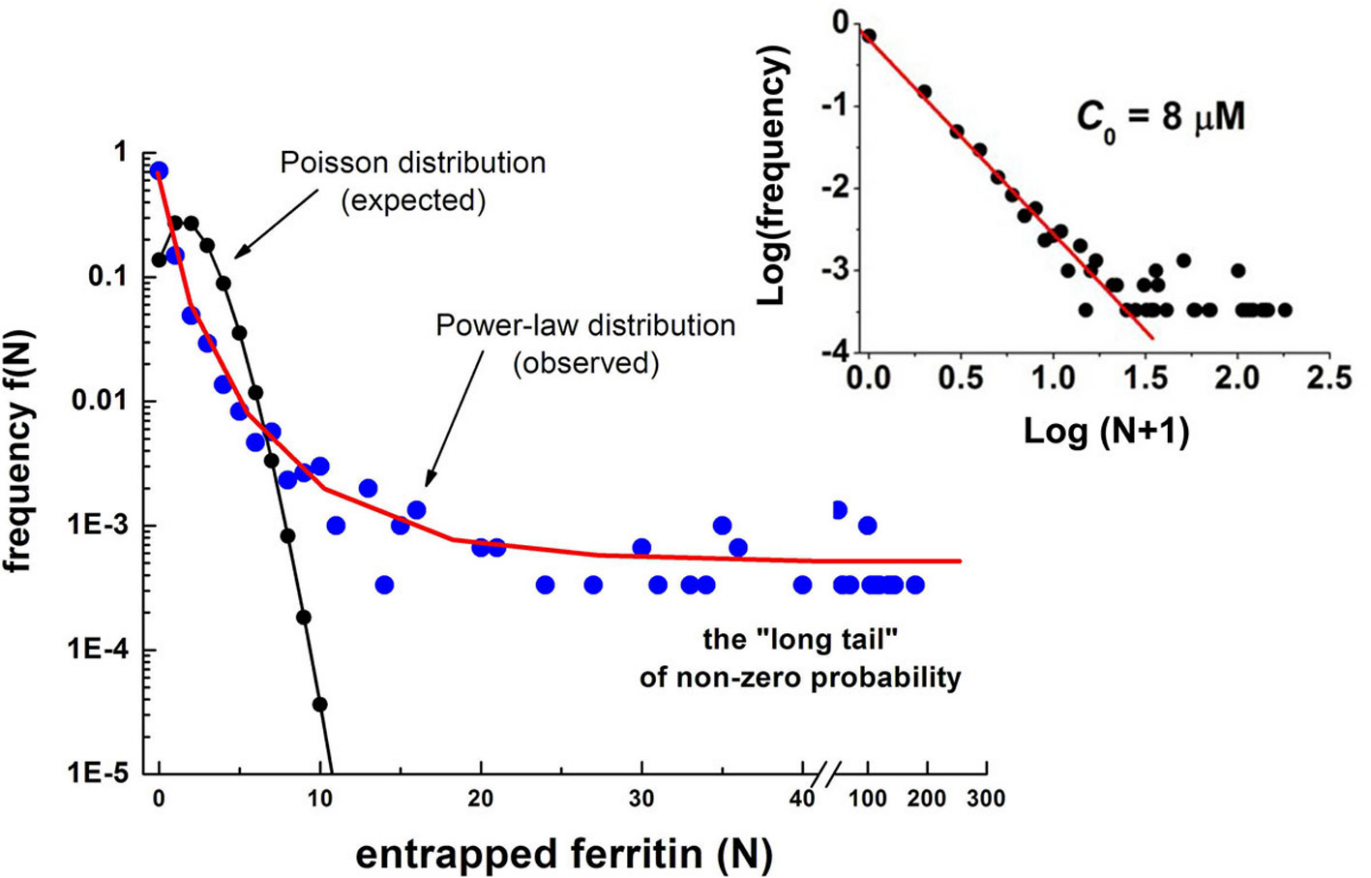
**Over-crowding effect**. Distribution of entrapped ferritin experimentally measured by means of electronic microscopy and theoretically forecasted Poisson distribution: the main divergence is given by the long tail of non-zero probability (redrawn from [31]).

This scenario is further complicated by recent - and very intriguing - findings which suggest that the encapsulation might not follow the Poisson statistics, but could be ruled by a power law. In particular, based on the observed occurrence of GFP production in 200 nm (diameter) vesicles, it was supposed that macromolecules could be indeed captured by closing lipid membranes with much higher a probability than the one calculated according to the Poisson distribution. To test this hypothesis, a thorough investigation was conducted on the physics of solute encapsulation by cryo- transmission electron microscopy. By directly counting the actual number of molecules per vesicle, it has been shown that when the protein ferritin [31] and ribosomes [32] are encapsulated inside lipid vesicles, most of the formed vesicles are actually empty, while a small minority (0.1-1%) contains an unexplained high number of solute molecules. This observation is not compatible with a Poisson entrapment model, but it follows a power law distribution (i.e., a distribution where the "long tail" predicts the occurrence of events which are very distant from the average behaviour (Figure 2). While these observations are a matter of fact, an explanation is still missing. No experimental studies are available as yet to ascertain whether a power law rules the encapsulation of multiple solutes, as in the PURE system. Further, there are no hints about the existence of solute- or membrane-specific interactions that might cause a deviation from the Poisson distribution.

Here we would like to investigate, by an *in silico *approach, the possible expectations -in terms of measurable protein synthesis yield- that follow from a set of hypotheses which are currently under experimental and theoretical scrutiny.

By following an approach philosophically similar to that of a previous work [33], where a stochastic simulation was used to test the viability of candidate minimal genome organisms, we would like to test the efficiency of different entrapment models to generate liposomes able to perform a complete protein synthesis. To do this, a specific simulator has been used, called QDC [34], based on Gillespie SSA algorithm, and able to control experimental parameters in a metabolic experiment and to deal with very large and very small number of molecules and volumes.

We think such approach can help advance the understanding of the implications of different hypotheses on solutes entrapment process. A simulation approach, in fact, can conveniently overcome the technical limits which hinder the "wet" study of these micro-compartmentalized systems, and predict clear-cut experimental observables, so providing a means to discriminate between alternative models.

## Results

### Formal specification of the PURE system

By assuming the simplifications declared in the Methods section, we derive a formal specification of the PURE system composed by 106 reactions, that best describe the behaviour of the system. The complete list of each simulated chemical species, reaction and kinetic coefficient is reported in the Additional File 1 (written in the QDC input syntax); we report in the Table 2 a selection of the most important kinetic constants, along with a concise description of their derivation.

**Table 2:** Derivation of kinetic constants

Reaction	Kinetic constant	Source
IF1 + R > RBS1	10^8 ^M^-1^s^-1^	[36]
RBS1 > IF1 + R	2.25 s^-1^	[36]
IX > eR + IF1 + IF2 + IF3 + GDP + Pi + BS	0.18 s^-1^	from frequency of new elongating ribosomes appearing on mRNA; [37]
IX > RBS + fMetRNA	0.23 s^-1^	from codon detection kinetics; [38]
EFtu + EFts > tuts	10^7 ^M^-1^s^-1^	[39]
tuts > EFtu + EFts	0.01 s^-1^	[39]
EX > eRa + EFtuGDP + Pi	15 s^-1^	from commonly accepted value of ribosome translocation rate (45 codons/s)
EX2 > EFg + GDP + Pi + eRp + tRNA	15 s^-1^	from commonly accepted value of ribosome translocation rate (45 codons/s)
EX2 > EFg + GDP + Pi + tRNA + eRSTOP	0.05 s^-1^	from length of GFP (~300 aminoacids) and ribosome translocation rate (45 codons/s)
aaSyn + ATP > AX	10^8 ^M^-1^s^-1^	diffusion limit
AX2 > aatRNA + Syn	0.84 s^-1^	from enzyme activities of the PURE system, as reported in [24]
AK + ATP > Y	10^6 ^M^-1^s^-1^	from Adenylate Kinase Km for ATP, as reported in [40]
Y > AK + ATP	40 s^-1^	from Adenylate Kinase Km for ATP, as reported in [40]
Y > AKP + ADP	300 s^-1^	[41]

See Additional file 2 for legend.

The model was used to perform stochastic simulations of the PURE system in a set volume, representing the internal volume of a liposome. Varying the system volume between the simulations allowed us to study the impact of this variable on the system dynamics. Even though the PURE system is well known and commercially available, to the best of our knowledge this is the first work where its dynamic behaviour is studied by means of a stochastic simulation.

### Simulating the protein synthesis in liposomes with the two entrapment models

Our primary target of study was translation efficiency. A measure of it was established as the time τ the system required in order to yield a fixed, arbitrary number of protein molecules per volume unit. We have computed this quantity for both entrapment models: the Poisson and the power-law, respectively.

#### τ (volume) for Poisson-distributed system species

For each simulated volume V, the mean number μ_i _of each of the 24 system species present in the reaction compartment at time zero was calculated from the initial concentrations of PURE system species as reported by [35].

μ_i _was set as the parameter defining the Poisson distribution for each i-th species, which was then used to generate 360 instances of the encapsulated system with stochastically distributed species.

**Figure 3:**
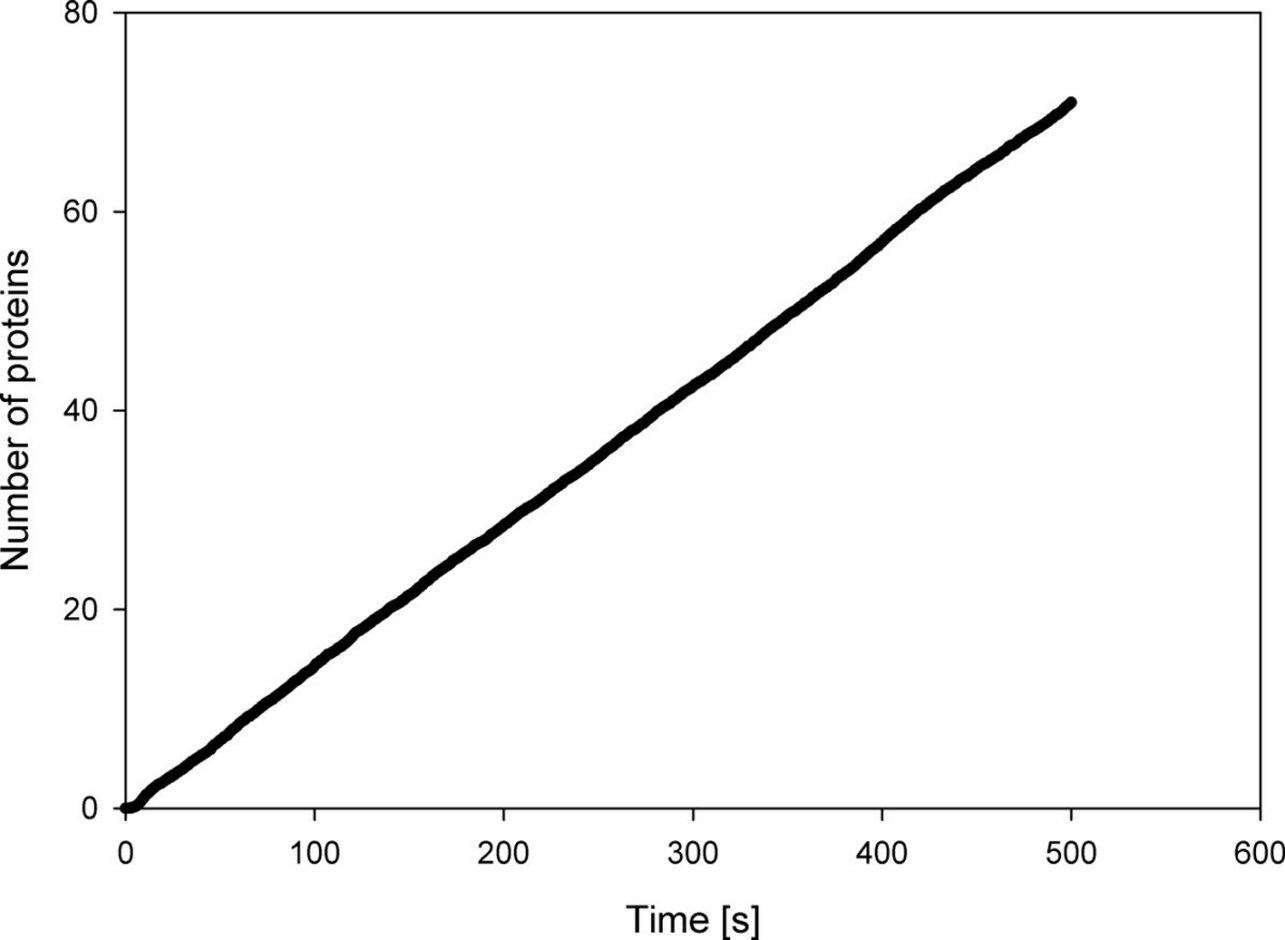
**Time course of protein synthesis in a 10^-16 ^L vesicle, Poisson model**. In the abscissa there is the time (in sec), where the ordinate shows the number of generated proteins.

The time course of the number of protein molecules was averaged instant-by-instant across all simulations, in order to cancel out stochastic effects not dependent on volume. The procedure was followed for each volume simulated, in a range from 10^-13 ^to 10^-18 ^L. Figure 3 graphically reports the result for a 10^-16 ^L compartment (vesicle outer diameter ca. 580 nm). The linear-like time course is due to a good translational efficiency, where the molecular machinery works regularly and the chemical resources are still available in relatively large quantities.

The data were used to derive the translation efficiency, as shown in Table 3.

**Table 3:** τ (volume) Poisson distribution

Volume (L)	Time to threshold (s)	Standard error (s)
10^-13^	68.5	< 0.01
5*10^-14^	68.5	< 0.01
10^-14^	68	0.01
5*10^-15^	67.5	0.02
10^-15^	67	0.07
5*10^-16^	67	0.09
4*10^-16^	66.5	0.09
3*10^-16^	66.5	0.1
2*10^-16^	67	0.1
10^-16^	71	0.11
9*10^-17^	74	0.33
8*10^-17^	80	0.69
7*10^-17^	90	1.11
6*10^-17^	101	2.34
5*10^-17^	120	4.01
4*10^-17^	149	7.85
3*10^-17^	161	15.06
2*10^-17^	219	27.71
10^-17^	> 1,000	n/a
5*10^-18^	n/a	n/a
10^-18^	n/a	n/a

**Figure 4:**
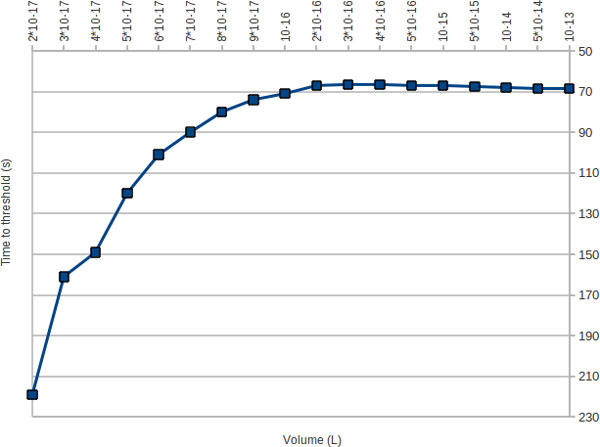
**τ(volume), Poisson**. In the abscissa is reported the vesicle volume (L); in the ordinate the time necessary to reach the synthesis threshold (in sec.); the orientation of this axis is reversed.

A peak of translation efficiency is revealed at around 3*10^-16 ^L, corresponding to liposomes of approximately 840 nm (Figure 4).

This maximum is reliable, as we repeated five runs of independent simulations, and its existence and position has been confirmed. The existence of this maximum is somewhat expected from a general consideration that sees the translational efficiency as the equilibrium between two opposite contributions. The former is given by pure kinetic effect: the smaller is the vesicle volume, the higher is the apparent kinetics of the chemical reactions of order higher than first. As almost all the reactions involved in the simulated PURE system are second order, their speed increases when the liposome size becomes smaller. The latter contribution derives from the Poisson entrapment process that makes highly probable that some necessary chemical species are missing when the liposome size becomes smaller. Therefore it is likely to expect that there is an optimal vesicle size that contains all the necessary chemical species in the minimum volume possible. Following our result, the translational efficiency decreases abruptly for vesicle smaller than the optimal size.

#### τ (volume) for power-law-distributed system species

For each simulated volume V, the corresponding "over-crowding concentration" c_s _was determined from the experimental data reported in [31] and graphically displayed in Figure 2. The number μ_s _of each solute in an "overcrowded" vesicle was calculated as μ_s _= c_s _N_A _V.

Solutes whose initial concentration according to [24] is higher than c_s _were not adjusted for the super-concentration effect, but left with their regular molecule numbers.

The frequency of the "overcrowded" vesicles was inferred from [31] and taken as between 0.1% - 1%, depending on super-concentration level, hence also on volume. So, the frequency was established as 1%, 0.2%, and 0.1% for vesicles of volume, respectively, 10^-16^, 5*10^-17^, and < = 10^-17 ^L (corresponding, respectively to vesicle diameters of ca. 580, 465 and < = 275 nm). This is a good choice to highlight the properties of power-law distribution, by avoiding, at the same time, to face a complex multivariate study.

The "non-overcrowded" vesicles were assigned a mean number μ*_i _*of species *i*, calculated as: μ*_i _*= [c*_i _*N_A _V (L_s _+ L_n_) - c_s _N_A _V L_s_]/L_n _, where c*_i _*is the regular concentration of species *i *as reported in [24] for the PURE system; L_s _is the number of overcrowded vesicles; L_n _is the number of non-overcrowded vesicles. The formula introduces a correction for the number of molecules already entrapped in the overcrowded vesicles, thus unavailable to be entrapped in the non-overcrowded liposomes.

For each volume V, in a range from 10^-16 ^to 10^-20 ^L (diameters from 580 to 35 nm), 1000 instances of the system were generated pursuant to the above-described procedure.

**Figure 5:**
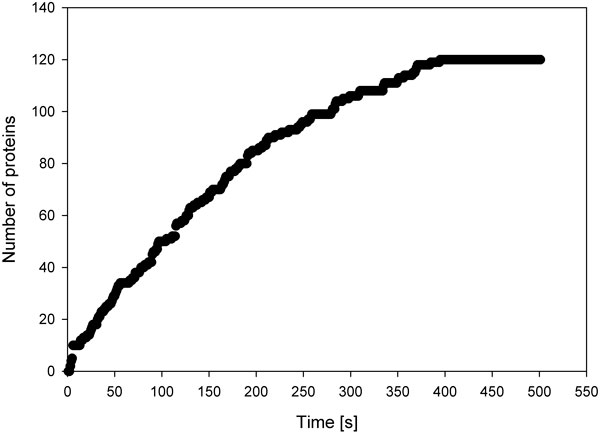
**Time course of protein synthesis in a 10^-17 ^L vesicle, power law model**. In the abscissa there is the time (in sec), where the ordinate shows the number of generated proteins.

Contrary to the Poisson model, the system reaches a protein production plateau within the simulation time limit (Figure 5). This is due to the rapid consumption of amino acids and energetic species by the over-concentrated PURE system enzymes. Results are presented in Table 4. The plateau and the general poor yield of the system made impractical the use of the same measure of efficiency as used in the Poisson series. In accordance to our aim to provide a means to discriminate between the Poisson and the power-law statistics in the wet-lab, the time required to reach the plateau, and the number of proteins at plateau per overcrowded vesicle, are given instead.

**Table 4:** τ (volume) power-law distribution

Volume (L)	Time to plateau (s)	Protein No. at plateau (per vesicle)
10^-16^	285	72.9
5*10^-17^	356	85
10^-17^	394	120
5*10^-18^	504	35
10^-18^	14	1
5*10^-19^	44	3
10^-19^	n/a	0
5*10^-20^	n/a	0

## Conclusions

The *in silico *analysis of the translational efficiency of liposomes entrapping the PURE system reveals that there is a size threshold of 10^-16 ^L below which the Poisson entrapment model is forecasted to give no detectable synthesis. The main experimental observable is therefore represented by the possible protein synthesis in liposomes of about 10^-17 ^L volume (275 nm diam.) or less. By entrapping the PURE system in liposomes of this size, with an mRNA specifying for a GFP protein, the detection of a fluorescence signal will indicate that the real distribution of solutes in liposome follows a power-law statistics and not a Poisson process. This experimental validation could improve our understanding about the basic mechanism underlying the relationship between membrane compartments formation and solute distribution within them.

## Methods

### Deriving a formal specification for the PURE system

In order to specify a simulation-amenable model, the biochemical transformations carried out by the PURE system components was decomposed into elementary reactions (first or second order reactions), whose deterministic kinetic coefficients have been harvested from the literature, or, when unavailable, deduced by global considerations of thermodynamic and/or chemical nature or by imposing an inner coherence to the system. The detail level chosen to specify the system is the result of two complementary needs: to maintain as low as possible the computational cost (that is very high for SSA-based simulators) and to explicitly represent any significant entity present in the system. The resulting main simplifications adopted are: amino acids, tRNAs, aminoacyl-tRNA synthetases, and translation Release Factors are modelled each as a single species representing its own entire class (e.g., one "general tRNA" in lieu of the 56 actual tRNAs of the PURE system). These simplifying assumptions allow reducing the number of chemical species involved in the transcription/translation reactions down to 24. Furthermore, every round of translation is so designed as to yield a complete, 300-amino acids long protein (which models the GFP). In other words there is no abortive translation. This is in good accord with wet-lab empirical observations. Autoradiography of synthesized protein after electrophoretic separation typically reveals only one major protein band [28].

### Perform a stochastic simulation of the biochemical species entrapped in the vesicles

Stochastic simulations have been performed thanks to QDC (Quick Direct-method controlled), a simulator based on the Gillespie's SSA algorithm (Direct-method version), described in the detail in [34]. The main reason to use such simulator is that it implements a series of checking tools that allowed us to verify the reliability of the obtained simulated data. In particular, it allows the use of very large/small quantities and it checks for possible overflow/underflow of variables that, given the volumes and concentrations used in our virtual experiments, are possible. Moreover, QDC outputs three files: one is containing the time course of the number of molecules of the various chemical species; one is containing the time course of the propensities of each chemical reaction, and the last file contains the effective firing rate of each reaction. By analysing these three files, it is possible to check for possible simulation artefacts or for possible system properties that can alter the simulation (e.g.: the possible stiffness of a system). We have deeply used these characteristics in order to rule out numerical errors or unsuitable descriptions of the biochemical system to be simulated.

### Generating the entrapment models

The Poisson distribution model has been generated by means of a laboratory made script in Python language that simply computes the average value for the molecules in a vesicle, given the vesicle volume and the concentration of the species in the original solution. Then the script calls the Poisson subroutine (available in the statistic library of Python language) that outputs a Poisson-distributed variable.

The power-law entrapment model has been generated by a direct inference on the experimental data, as recalled in the Results and discussion section.

## Competing interests

The authors declare that they have no competing interests.

## Authors' contributions

PLL and PS supplied the experimental data and checked the model consistency and reliability from an experimental point of view. LLO and RM designed the theoretical model and performed the simulations. All the Authors have contributed in the data analysis and manuscript preparation.

## Supplementary Material

Additional file 1 **Formal specification of the PURE system in the QDC input language**: This file contains, in ASCII format, an example of the complete input file for a PURE system containing liposomes. Accordingly with QDC syntax, the file is divided into blocks: the first contains the declaration of the chemical species present in the system; the second block contains the vesicle volume; the third block contains the chemical reactions that can take place (each is described as: "k, R1 + R2 > P1 + P2", where k is the deterministic kinetic coefficient, R1 and R2 are the reagents; P1 and P2 are the products); the fourth (and last) block contains the initial number of molecules for each chemical species. Click here for file

Additional file 2 **This file contains the list of each chemical species declared in the file puresys.txt along with the description of which real chemical species it is referred to**. Click here for file

## References

[B1] Orgel L RNA catalysis and the origin of life J Theor Biol 1986 123 127 149 10.1016/S0022-5193(86)80149-7 2442564

[B2] Morowitz HJ The Beginnings of Cellular Life: Metabolism Recapitulates Biogenesis 1992 New Haven, Yale University Press

[B3] Szostak JW Bartel DP Luisi PL Synthesizing life Nature 2001 409 387 390 10.1038/35053176 11201752

[B4] Luisi PL Ferri F Stano P Approaches to semi-synthetic minimal cells: a review Naturwissenschaften 2006 93 1 13 10.1007/s00114-005-0056-z 16292523

[B5] Lorsch JR Chance and necessity in the selection of nucleic acid catalysts Acc Chem Res 1996 29 103 110 10.1021/ar9501378 11539421

[B6] Wächtershäuser G The origin of life and its methodological challenge J Theor Biol 1997 187 483 94 10.1006/jtbi.1996.0383 9299293

[B7] Luisi PL The Emergence of Life, From Chemical Origins to Synthetic Biology 2006 Cambridge, Cambridge University Press

[B8] Stano P Carrara P Kuruma Y Souza T Luisi PL Compartmentalized reactions as a case of soft-matter biotechnology: Synthesis of proteins and nucleic acids inside lipid vesicles J Mater Chem 2011 21 18887 18902 10.1039/c1jm12298c

[B9] Walde P Wick R Fresta M Mangone A Luisi PL Autopoietic self-reproduction of fatty acid vesicles J Am Chem Soc 1994 116 11649 11654 10.1021/ja00105a004

[B10] Luisi PL Autopoiesis: a review and a reappraisal Naturwissenschaften 2003 90 49 59 12590297 10.1007/s00114-002-0389-9

[B11] Luisi PL Rasi PS Mavelli F A possible route to prebiotic vesicle reproduction Artif Life 2004 10 297 308 10.1162/1064546041255601 15245629

[B12] Bachmann PA Luisi PL Lang J Autocatalytic self-replicating micelles as models for prebiotic structures Nature 1992 357 57 59 10.1038/357057a0

[B13] Hanczyc MM Szostak JW Replicating vesicles as models of primitive cell growth and division Curr Opin Chem Biol 2004 8 660 664 10.1016/j.cbpa.2004.10.002 15556412

[B14] Zhu TF Szostak JW Coupled growth and division of model protocell membranes J Am Chem Soc 2009 131 5705 5713 10.1021/ja900919c 19323552 PMC2669828

[B15] Bloechliger E Blocher M Walde P Luisi PL Matrix Effect in the Size Distribution of Fatty Acid Vesicles J Phys Chem 1998 102 10383 10390 10.1021/jp981234w

[B16] Rasi S Mavelli F Luisi PL Cooperative micelle binding and matrix effect in oleate vesicle formation J Phys Chem B 2003 107 14068 14076 10.1021/jp0277199

[B17] Stano P Wehrli E Luisi PL Insights on the oleate vesicles self-reproduction J Physics: Cond Matter 2006 18 S2231 S2238 10.1088/0953-8984/18/33/S37

[B18] Yu W Sato K Wakabayashi M Nakaishi T Ko-Mitamura EP Shima Y Urabe I Yomo T Synthesis of functional protein in liposome J Biosci Bioeng 2001 92 590 593 16233152 10.1263/jbb.92.590

[B19] Oberholzer T Meyer E Amato I Lustig A Monnard PA Enzymatic reactions in liposomes using the detergent-induced liposome loading method Biochim Biophys Acta 1999 1416 57 68 10.1016/S0005-2736(98)00210-7 9889319

[B20] Fischer A Franco A Oberholzer T Giant vesicles as microreactors for enzymatic mRNA synthesis Chembiochem 2002 3 409 417 10.1002/1439-7633(20020503)3:5<409::AID-CBIC409>3.0.CO;2-P 12007174

[B21] Walde P Ichikawa S Enzymes inside lipid vesicles: preparation, reactivity and applications Biomol Eng 2001 18 143 77 10.1016/S1389-0344(01)00088-0 11576871

[B22] Noireaux V Libchaber A A vesicle bioreactor as a step toward an artificial cell assembly PNAS 2004 101 17669 17674 10.1073/pnas.0408236101 15591347 PMC539773

[B23] Luisi PL Toward the Engineering of Minimal Living Cells Anat Record 2002 268 208 214 10.1002/ar.10155 12382319

[B24] Shimizu Y Inoue A Tomari Y Suzuki T Yokogawa T Nishikawa K Ueda T Cell-free translation reconstituted with purified components Nat Biotechnol 2001 19 751 755 10.1038/90802 11479568

[B25] Sunami T Sato K Matsuura T Tsukada K Urabe I Yomo T Femtoliter compartment in liposomes for in vitro selection of proteins Analyt Biochem 2006 357 128 136 10.1016/j.ab.2006.06.040 16889743

[B26] Murtas G Kuruma Y Bianchini P Diaspro A Luisi PL Protein synthesis in liposomes with a minimal set of enzymes Bioch Biophys Res Comm 2007 363 12 17 10.1016/j.bbrc.2007.07.201 17850764

[B27] Souza T Stano P Luisi PL The minimal size of liposome-based model cells brings about a remarkably enhanced entrapment and protein synthesis ChemBioChem 2009 10 1056 1063 10.1002/cbic.200800810 19263449

[B28] Kuruma Y Stano P Ueda T Luisi PL A synthetic biology approach to the construction of membrane proteins in semi-synthetic minimal cells Biochim Biophys Acta 2009 1788 567 574 10.1016/j.bbamem.2008.10.017 19027713

[B29] Kita H Matsuura T Sunami T Hosoda K Ichinashi N Tsukada K Urabe I Yomo T Replication of Genetic Information with Self-Encoded Replicase in Liposomes ChemBioChem 2008 9 2403 2410 10.1002/cbic.200800360 18785673

[B30] Saito H Kato Y Le Berre M Yamada A Inoue T Yosikawa K Baigl D Time-Resolved Tracking of a Minimum Gene Expression System Reconstituted in Giant Liposomes ChemBioChem 2009 10 1640 1643 10.1002/cbic.200900205 19533718

[B31] Luisi PL Allegretti M Pereira de Souza T Steiniger F Fahr A Stano P Spontaneous protein crowding in liposomes: a new vista for the origin of cellular metabolism Chembiochem 2010 11 1989 1992 10.1002/cbic.201000381 20806308

[B32] Souza T Steiniger F Stano P Fahr A Luisi PL Spontaneous crowding of ribosomes and proteins inside vesicles: A possible mechanism for the origin of cell metabolism ChemBioChem 2011 in press doi: 10.1002/cbic.201100306 10.1002/cbic.201100306 21830290

[B33] Chiarugi D Degano P Marangoni R A Computational Approach to the Functional Screening of Genomes PLoS Comp Biol 2007 3 e174 10.1371/journal.pcbi.0030174 PMC1994977 17907794

[B34] Cangelosi D Fabbiano S Felicioli C Freschi L Marangoni R QDC (Quick Direct-method Controlled): a simulator of metabolic experiments IET Systems Biol 2011 in press

[B35] Shimizu Y Kanamori T Ueda T Protein synthesis by pure translation systems Methods 2005 36 299 304 10.1016/j.ymeth.2005.04.006 16076456

[B36] Kierzek AM Zaim J Zielenkiewicz P The effect of transcription and translation initiation frequencies on the stochastic fluctuations in prokaryotic gene expression J Biol Chem 2001 276 8165 8172 10.1074/jbc.M006264200 11062240

[B37] Kennell D Riezman H Transcription and translation initiation frequencies of the *Escherichia coli *lac operon J Mol Biol 1977 114 1 21 10.1016/0022-2836(77)90279-0 409848

[B38] Gromadski KB Rodnina MV Kinetic determinants of high-fidelity tRNA discrimination on the ribosome Mol Cell 2004 13 191 200 10.1016/S1097-2765(04)00005-X 14759365

[B39] Wieden HJ Mercier E Gray J Steed B Yawney D A combined molecular dynamics and rapid kinetics approach to identify conserved three-dimensional communication networks in elongation factor Tu Biophys J 2010 99 3735 3743 10.1016/j.bpj.2010.10.013 21112298 PMC2998634

[B40] Glaser P Prescecan E Delepierre M Surewicz WK Mantsch HH Bârzu O Gilles AM Zinc, a novel structural element found in the family of bacterial adenylate kinases Biochemistry 1992 31 3038 3043 10.1021/bi00127a002 1554691

[B41] Ramotar K Pickard MA AMP metabolism by the marine bacterium Vibrio (Benecka) natriegens: purification and properties of adenylate kinase Can J Microbiol 1981 27 1053 1059 10.1139/m81-164

[B42] Stano P Kuruma Y Souza TP Luisi PL Biosynthesis of proteins inside liposomes Methods in Molecular Biology 2010 606 127 145 20013395 10.1007/978-1-60761-447-0_11

